# Sex-stratified association between systemic immune-inflammation index and gallstone risk: a cross-sectional study in a Chinese adult population

**DOI:** 10.3389/fpubh.2026.1923451

**Published:** 2026-10-05

**Authors:** Yajie Teng, Daiyi Zhang, Yingxue Yang, Lijuan Qian, Qinghua Wang

**Affiliations:** 1 Department of Gastroenterology, The First People's Hospital of Kunshan, Suzhou, Jiangsu, China; 2 Health Management Center, The First Affiliated Hospital of Soochow University, Suzhou, Jiangsu, China; 3 Department of Gastroenterology, The First Affiliated Hospital of Soochow University, Suzhou, Jiangsu, China

**Keywords:** Chinese population, cross-sectional study, gallstone, sex-specific, systemic immune-inflammation index

## Abstract

**Introduction:**

Gallstone disease is a prevalent biliary condition with rising incidence in China, driven by changes in lifestyle, metabolism, and inflammation. The systemic immune-inflammation index (SII), a composite marker reflecting immune and inflammatory status, has been linked to various chronic diseases. However, its association with gallstone in the general Chinese population remains unclear. This study aimed to investigate the relationship between SII and gallstone risk in a large adult cohort.

**Methods:**

A cross-sectional analysis was conducted among 55,241 adults undergoing health screening. Gallstone was diagnosed via abdominal ultrasonography. SII was log-transformed (Ln-SII) to correct for skewness. Logistic regression models assessed the association between Ln-SII and gallstone risk with progressive adjustments. Smooth curve fitting and two-piece wise linear regression were used to explore potential nonlinear associations. Subgroup analyses were performed by sex.

**Results:**

Gallstone was identified in2,606 participants (4.72%). After full adjustment, higher Ln SII was significantly associated with increased gallstone risk (OR = 1.10, 95% CI: 1.01–1.21, *p* = 0.0372). In sex-stratified analyses, a significant linear association was found in females (OR = 1.21, 95% CI: 1.06–1.39, *p* = 0.0059), whereas a nonlinear association emerged in males, with increased risk only above an Ln-SII threshold of 6.52. Despite these sex-specific patterns, no statistically significant interaction between sex and SII was observed.

**Conclusion:**

Higher Ln SII was associated with increased gallstone risk, and sex-stratified analyses revealed distinct association patterns. However, no significant interaction between sex and SII was observed, suggesting that these differences may not represent effect modification. Further studies are needed to clarify the underlying mechanisms.

## Introduction

Gallstones are among the most prevalent gastrointestinal disorders, with a prevalence of approximately 20% in several Western countries, including the United States and Europe (1, 2). Even in populations with the lowest incidence, such as those in Asia and Africa, the prevalence ranges from 5 to 20% (3). Furthermore, with the increasing intake of high-calorie, high-carbohydrate, and low-fiber diets (nutritional excess) coupled with rising sedentary lifestyles, the prevalence of gallstone disease has been steadily increasing (4). While most gallbladder stones are asymptomatic or cause only mild discomfort, acute episodes can have potentially life-threatening consequences, including acute cholangitis, acute biliary pancreatitis, acute cholecystitis, and an elevated risk of gallbladder cancer (5–7). The need for cholecystectomy or treatment of these complications significantly contributes to the global healthcare burden.

Previous studies have identified several risk factors for gallstone formation, including age, sex, female reproductive status, obesity, cardiovascular disease, microbiome alterations, glucose metabolism, systemic inflammation and various environmental exposures (8, 9). The Systemic Immune-Inflammation Index (SII), which is calculated by multiplying the platelet count by the neutrophil count and dividing by the lymphocyte count, has emerged as a novel and reliable marker for comprehensively assessing the systemic levels of immunity and inflammation (10). Numerous studies have demonstrated the association between SII and a variety of diseases, including various cancers such as hepatocellular carcinoma (10) and colorectal cancer (11), diabetes (12), cardiovascular diseases (13), hepatic steatosis (14), and gallstones (15). Increasing evidence suggests that systemic inflammation may contribute to gallstone development through alterations in gallbladder function and bile homeostasis (16). Chronic inflammatory responses can promote the secretion of pro-inflammatory cytokines, such as interleukin-6 (IL-6) and tumor necrosis factor-alpha (TNF-α), which may influence hepatic lipid metabolism, cholesterol secretion into bile, and gallbladder epithelial function, thereby facilitating cholesterol crystal nucleation and gallstone formation (17, 18). As SII integrates neutrophil, lymphocyte, and platelet counts, it may provide insight into the inflammatory and immune pathways involved in gallstone pathogenesis. Neutrophils are key mediators of innate inflammation and can promote tissue injury through cytokine release, oxidative stress, and neutrophil extracellular trap (NET) formation, potentially contributing to gallbladder mucosal inflammation and cholesterol crystal aggregation (19, 20). Lymphocytes play essential roles in immune regulation, and reduced lymphocyte levels may reflect impaired immune homeostasis and a shift toward chronic low-grade inflammation, which is commonly associated with metabolic abnormalities and gallstone risk (21). Platelets, beyond their classical role in coagulation, participate in inflammatory regulation by interacting with immune cells and releasing inflammatory mediators, potentially amplifying local and systemic inflammatory responses (22). Therefore, an elevated SII may represent a biological link between systemic inflammation, altered bile metabolism, and gallstone formation. Previous studies have established the relationship between inflammatory markers and gallstones in the middle-aged and young adult populations in the United States (23, 24). However, there is a lack of further exploration in the Chinese population. Therefore, this study aims to evaluate the association between SII and gallstone disease in a large Chinese adult population.

## Methods

### Study population

This retrospective study analyzed the medical records of 55,241 adult individuals who underwent routine health examinations—including blood tests and abdominal ultrasonography—at the First Affiliated Hospital of Soochow University between January and December 2024. All participants completed a standardized questionnaire designed to collect demographic characteristics, medical history, and lifestyle factors. The collected information included smoking status (categorized as current, former, or never), alcohol drinking (categorized as current, former, or never), and daily physical activity (defined as engaging in at least 150 min of moderate-intensity aerobic exercise or at least 75 min of vigorous-intensity activity per week). Medical histories of diabetes and hypertension were also assessed. Diabetes mellitus was defined based on a self-reported physician diagnosis, current use of glucose-lowering medication, or laboratory evidence consistent with established diagnostic criteria. Hypertension was defined as a self-reported physician diagnosis, current use of anti-hypertensive medication, or measured blood pressure meeting the diagnostic threshold. Subsequently, all participants underwent a comprehensive physical examination. A total of 5,476 participants were excluded due to missing key variables, a documented history of severe hepatobiliary diseases, pregnancy, or poorly controlled chronic diseases (including uncontrolled diabetes mellitus, hypertension, cardiovascular diseases, and renal dysfunction). Severe hepatobiliary diseases, including liver cirrhosis, liver cancer, viral hepatitis, cholangitis, biliary obstruction, and other clinically significant hepatic or biliary disorders, were identified based on questionnaire responses, previous medical records, physician diagnoses, and relevant diagnostic codes. A detailed flow diagram of the participant inclusion and exclusion process is presented in Figure 1.

**Figure 1:**
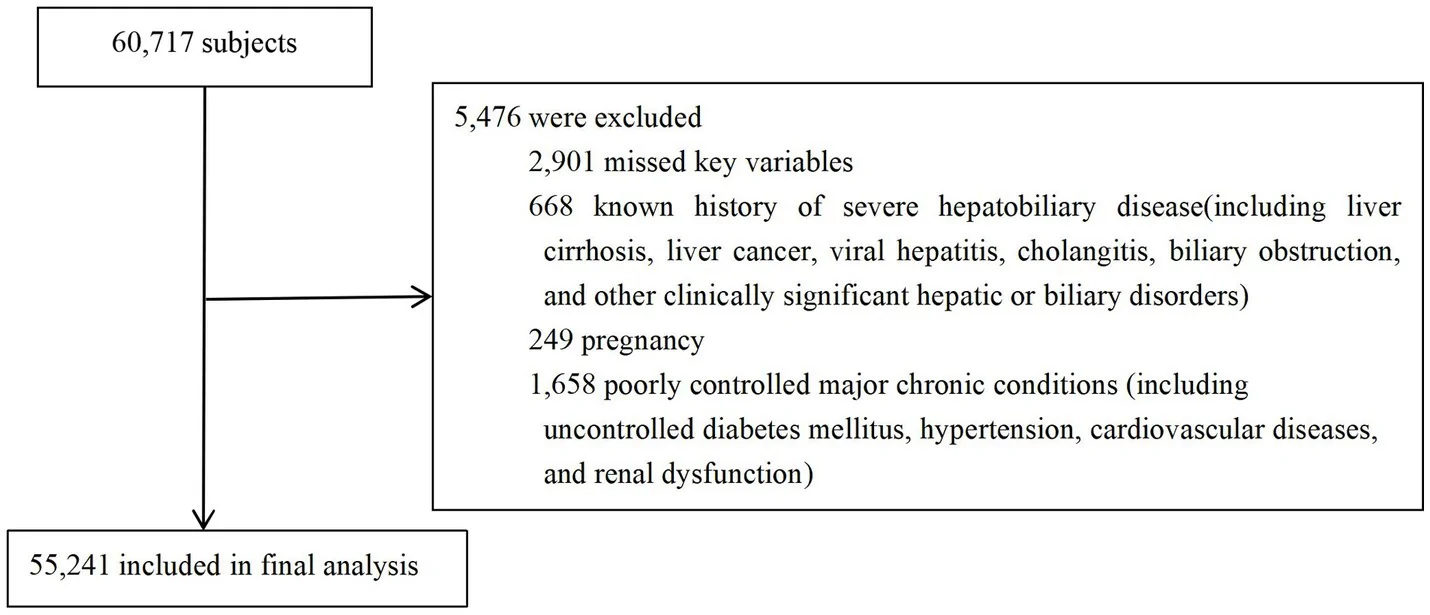
Flow diagram of patient inclusion and exclusion.

### Ethics approval and consent to participate

The study protocol was reviewed and approved by the Ethics Committee of the First Affiliated Hospital of Soochow University (Approval No. [2025] Lun Approval No. 702) and conducted in accordance with the Declaration of Helsinki. Due to the retrospective nature of the study and the use of anonymized secondary data, the requirement for informed consent from participants was waived.

### Laboratory testing and abdominal ultrasound examination

Fasting venous blood samples were collected from the antecubital vein of each participant after at least 12 h of fasting, typically in the morning. White blood cell (WBC) count, platelet (PLT) count, neutrophil (NE) count, and lymphocyte (L) count were measured using a Sysmex XN automated blood cell analyzer (Sysmex Corporation, Japan). The SII index was calculated as follows: peripheral platelet count (PLT, ×10^9^/L) × absolute neutrophil count (NE, ×10^9^/L) / absolute lymphocyte count (L, ×10^9^/L).

The distribution of SII was assessed before analysis and showed significant positive skewness. Therefore, SII was logarithmically transformed using the natural logarithm to approximate a normal distribution, reduce heteroscedasticity, and improve the robustness and interpretability of regression models. Serum levels of aspartate aminotransferase (AST), alanine aminotransferase (ALT), *γ*-glutamyl transferase (GGT), total cholesterol (TC), triglycerides (TG), high-density lipoprotein cholesterol (HDL-C), low-density lipoprotein cholesterol (LDL-C), uric acid (UA), fasting plasma glucose (FPG), and homocysteine (Hcy) were quantified using standard enzymatic techniques on the LABOSPECT 008 α automated biochemical analyzer (HITACHI, Japan). Abdominal ultrasound examinations were performed with an ACUSON Redwood color Doppler ultrasound system (Siemens, Germany). Gallstone status was assessed by abdominal ultrasonography during routine health examinations. Gallstones were defined as the presence of discrete hyperechoic structures within the gallbladder lumen with posterior acoustic shadowing and/or positional mobility with changes in body position. Gallbladder sludge, gallbladder polyps, and other non-calculous biliary abnormalities were not considered gallstones and were excluded from the diagnosis. All ultrasound examinations were performed by experienced sonographers and reviewed by senior sonographers according to standardized diagnostic criteria. In cases of diagnostic uncertainty, the final determination was made after discussion and consensus among senior sonographers. Regular quality control procedures were implemented to ensure the consistency and reliability of ultrasound-based diagnoses.

### Statistical analysis

All statistical analyses were conducted using R software (version 3.6.2). Demographic characteristics and potential risk factors for gallstone were expressed as mean ± standard deviation (Mean ± SD) for continuous variables or frequency (percentage) for categorical variables. Continuous variables were compared using the Kruskal–Wallis test, while categorical variables were analyzed using Fisher’s exact test. To evaluate multicollinearity in the fully adjusted model, variance inflation factors (VIFs) were calculated. A VIF value >5 was considered indicative of potential multicollinearity issues. Multivariable logistic regression models were used to evaluate the association between Ln-SII and gallstone prevalence after adjusting for potential confounders. Three regression models were constructed with progressive adjustments. Model I was adjusted for age, sex, body mass index (BMI), and other potential confounding factors, including hypertension, diabetes, daily exercise, smoking status, and alcohol drinking status. Model II was further adjusted for lipid-related indicators, including triglycerides (TG), high-density lipoprotein cholesterol (HDL-C), and low-density lipoprotein cholesterol (LDL-C), in addition to the covariates included in Model I. To explore the potential non-linear association between Ln-SII and gallstone prevalence, a generalized additive model (GAM) with penalized smoothing splines was applied. The smooth function was fitted after adjustment for covariates included in the multivariable logistic regression model. The optimal degree of smoothness was automatically determined based on the data using the smoothing parameter estimated by the GAM algorithm. A two-piece wise linear regression model was applied to further evaluate the potential threshold effect. The inflection point was determined using a maximum likelihood estimation approach. Specifically, a recursive algorithm was used to examine a series of candidate threshold values within the observed range of Ln-SII. For each potential threshold, piece wise linear regression models were fitted, and the optimal threshold value was identified based on the maximum likelihood criterion, corresponding to the model with the best fit.

## Results

### Baseline characteristics of participants

A total of 55,241 participants were included in the analysis, among whom 2,606 (4.72%) were diagnosed with gallstone disease. The baseline characteristics of participants with and without gallstones are summarized in Table 1. Compared with participants without gallstones, those with gallstones were significantly older (51.33 ± 12.74 vs. 43.16 ± 12.38 years, *p* < 0.001) and had higher BMI values (25.13 ± 3.59 vs. 23.96 ± 3.49 kg/m^2^, *p* < 0.001). A higher proportion of males were observed in the gallstone group (58.71% vs. 50.23%, *p* < 0.001). Additionally, participants with gallstones had higher prevalence of hypertension (23.87% vs. 11.51%, *p* < 0.001) and diabetes (7.37% vs. 3.26%, *p* < 0.001). Significant differences were also observed in several laboratory parameters, including elevated levels of CRP (*p* = 0.002), ALT (*p* < 0.001), AST (*p* = 0.002), TG (*p* < 0.001), Hcys (*p* = 0.013), UA (*p* < 0.001), and FBG (*p* < 0.001), as well as decreased HDL-C (*p* < 0.001). No significant differences were observed in total cholesterol, LDL-C, or Ln-SII index (*p* = 0.425).

**Table 1:** Baseline characteristics of participants with and without gallstones.

Characteristic	Without gallstones	With gallstones	*p*-value
*N*	52,635	2,606	
Age	43.16 ± 12.38	51.33 ± 12.74	<0.001
Male	26,437 (50.23%)	1,530 (58.71%)	<0.001
BMI (kg/m^2^)	23.96 ± 3.49	25.13 ± 3.59	<0.001
Daily exercise			0.808
Yes	13,159 (25.00%)	646 (24.79%)	
No	39,476 (75.00%)	1960 (75.21%)	
Smoking			<0.001
Current or ever	9,808 (18.63%)	638 (24.48%)	
Never	42,827 (81.37%)	1968 (75.52%)	
Alcohol drinking			0.002
Current or ever	8,378 (19.52%)	474 (18.19%)	
Never	44,257 (84.08%)	2,132 (81.81%)	
WBC (×10^9^/L)	6.13 ± 1.56	6.24 ± 1.61	<0.001
NEU (×10^9^/L)	3.39 ± 1.15	3.49 ± 1.20	<0.001
PLT (×10^9^/L)	241.21 ± 56.50	234.64 ± 60.10	<0.001
LYM (×10^9^/L)	2.12 ± 0.62	2.10 ± 0.63	0.038
CRP (mg/L)	2.30 ± 2.29	2.73 ± 2.71	0.002
ALT (U/L)	23.85 ± 21.30	25.41 ± 19.51	<0.001
AST (U/L)	19.97 ± 11.18	20.66 ± 9.86	0.002
TC (mmol/L)	4.96 ± 0.91	4.96 ± 0.96	0.727
TG (mmol/L)	1.54 ± 1.23	1.75 ± 1.34	<0.001
HDL-C (mmol/L)	1.32 ± 0.33	1.25 ± 0.31	<0.001
LDL-C (mmol/L)	2.89 ± 0.77	2.91 ± 0.81	0.224
Hcys (umol/L)	10.31 ± 5.55	11.55 ± 10.81	0.013
UA (umol/L)	335.05 ± 88.11	346.24 ± 85.04	<0.001
FBG (mmol/L)	5.10 ± 0.98	5.36 ± 1.15	<0.001
Diabetes			<0.001
Yes	1715 (3.26%)	192 (7.37%)	
No	50,920 (96.74%)	2,414 (92.63%)	
Hypertension			<0.001
Yes	6,057 (11.51%)	622 (23.87%)	
No	46,578 (88.49%)	1984 (76.13%)	
SII index	411.19 ± 205.35	419.04 ± 220.65	0.058
Ln-SII index	5.92 ± 0.45	5.92 ± 0.48	0.425

Data are presented as mean ± standard deviation for continuous variables or frequency (percentage) for categorical variables. Continuous variables were compared using the Kruskal–Wallis test; categorical variables were compared using Fisher’s exact test. BMI, body mass index; ALT, alanine aminotransferase; AST, aspartate aminotransferase; TC, total cholesterol; TG, triglyceride; HDL-C, high-density lipoprotein cholesterol; LDL-C, low-density lipoprotein cholesterol; Hcys, homocysteine; UA, uric acid; FBG, fasting blood glucose; SII index, systemic immune-inflammatory index; Ln-SII index, natural logarithm-transformed SII.

### Association between Ln-SII and gallstone prevalence

The logistic regression analysis assessing the association between the Ln-SII and gallstone prevalence is shown in Table 2. In the unadjusted model, Ln-SII was not significantly associated with gallstone risk (odds ratio (OR) = 1.04, 95% CI:0.95–1.13, *p* = 0.4247). However, after adjustment for sex, age, BMI, hypertension, diabetes, daily exercise, smoking, and alcohol drinking status (Model I), the association became significant (OR = 1.11, 95% CI: 1.02–1.22, *p* = 0.0196). The fully adjusted Model II, which further included TG, HDL-C, LDL-C, confirmed this association (OR = 1.10, 95% CI: 1.01–1.21, *p* = 0.0372).

**Table 2:** Logistic regression results for the association of Ln-SII with gallstone prevalence.

With gallstones	Crude model		Model I		Model II	
	OR, (95% CI)	*p*-value	OR, (95% CI)	*p*-value	OR, (95% CI)	*p*-value
Ln-SII index	1.04 (0.95, 1.13)	0.4247	1.11 (1.02, 1.22)	0.0196	1.10 (1.01, 1.21)	0.0372

Model I is adjusted for: Sex; age; BMI; hypertension; diabetes; daily exercise; smoking; alcohol drinking.

Model II is adjusted for: Sex; age; BMI; hypertension; diabetes; daily exercise; smoking; alcohol drinking; TG; HDL-C; LDL-C.

To evaluate potential multicollinearity among covariates in the fully adjusted model, variance inflation factors (VIFs) were calculated (Table 3). All VIF values ranged from 1.0 to 1.8 and were below the conventional threshold of 5, indicating no significant multicollinearity among the included variables.

**Table 3:** VIF-based multicollinearity diagnostics for covariates included in adjusted models.

Variable	VIF
Ln-SII index	1
Sex	1.8
Age	1.3
BMI	1.5
Hypertension	1.3
Diabetes	1.1
Daily exercise	1.1
Smoking	1.5
Alcohol drinking	1.4
HDL-C	1.6
LDL-C	1.1
TG	1.3

All values were below the conventional threshold of 5.

### The sex subgroup analysis for association between Ln-SII and gallstone prevalence

Sex-stratified analyses were conducted to assess potential effect modification by sex (Table 4). In the male subgroup, no significant association was observed between Ln-SII and gallstone prevalence in either the unadjusted or adjusted models. Specifically, in Model I, Ln-SII was not significantly associated with gallstone prevalence (OR: 1.03; 95% CI: 0.91–1.16; *p* = 0.6799). After additional adjustment for lipid-related parameters in Model II, the association remained non-significant (OR: 1.02; 95% CI: 0.90–1.15; *p* = 0.7913). In contrast, a significant positive association between Ln-SII and gallstone prevalence was observed among females after multivariable adjustment. In the unadjusted model, the association was not statistically significant (OR: 1.08; 95% CI: 0.95–1.23; *p* = 0.2354). However, after adjustment for age, BMI, hypertension, diabetes, daily exercise, smoking status, and alcohol drinking status (Model I), higher Ln-SII levels were significantly associated with increased odds of gallstone prevalence (OR: 1.23; 95% CI: 1.07–1.41; *p* = 0.0033). This association remained robust after further adjustment for lipid-related parameters, including triglycerides (TG), high-density lipoprotein cholesterol (HDL-C), and low-density lipoprotein cholesterol (LDL-C), in Model II (OR: 1.21; 95% CI: 1.06–1.39; *p* = 0.0059). Although the magnitude and direction of the associations appeared to differ between males and females, formal interaction analyses did not demonstrate a statistically significant interaction between sex and Ln-SII. The *p*-values for interaction were 0.8954 in the unadjusted model, 0.0552 in Model I, and 0.0609 in Model II.

**Table 4:** Sex-stratified logistic regression analyses of the association between Ln-SII and gallstone prevalence.

With gallstones	Crude model		Adjust I		Adjust II	
	OR, (95% CI)	*p*-value	OR, (95% CI)	*p*-value	OR, (95% CI)	*p*-value
Male						
Ln-SII index	1.07 (0.95, 1.20)	0.2657	1.03 (0.91, 1.16)	0.6799	1.02 (0.90, 1.15)	0.7913
Female						
Ln-SII index	1.08 (0.95, 1.23)	0.2354	1.23 (1.07, 1.41)	0.0033	1.21 (1.06, 1.39)	0.0059
P interaction	0.8954		0.0552		0.0609	

Model I is adjusted for: age; BMI; hypertension; diabetes; daily exercise; smoking; alcohol drinking.

Model II is adjusted for: age; BMI; hypertension; diabetes; daily exercise; smoking; alcohol drinking; TG; HDL-C; LDL-C.

### Non-linear exploratory analysis of the association between Ln-SII and gallstone prevalence

To further characterize the potential nonlinear relationship between the natural logarithm-transformed Ln-SII and the prevalence of gallstones, smoothing spline curve fitting was performed in the overall population and stratified by sex to examine the consistency of the observed associations. As shown in Figure 2, a nonlinear association between Ln-SII and gallstone prevalence was observed in the overall population, with a similar pattern detected among males. In contrast, the association appeared to be more linear among females. In the generalized additive model (GAM), the smooth term for Ln-SII showed a borderline nonlinear association in the overall population (edf = 2.82, Ref.df = 3.65, *χ*^2^ = 7.81, *p* = 0.0788; UBRE = −0.64; *N* = 52,402). A similar nonsignificant pattern was observed in the male subgroup (edf = 1.74, Ref.df = 2.24, *χ*^2^ = 1.00, *p* = 0.6125; UBRE = −0.60; *N* = 27,080). However, among females, the smooth term for Ln-SII was statistically significant (edf = 1.01, Ref.df = 1.01, *χ*^2^ = 7.60, *p* = 0.0061; UBRE = −0.68; *N* = 25,322), suggesting a significant association between Ln-SII and gallstone prevalence (Supplementary Table S1).

**Figure 2:**
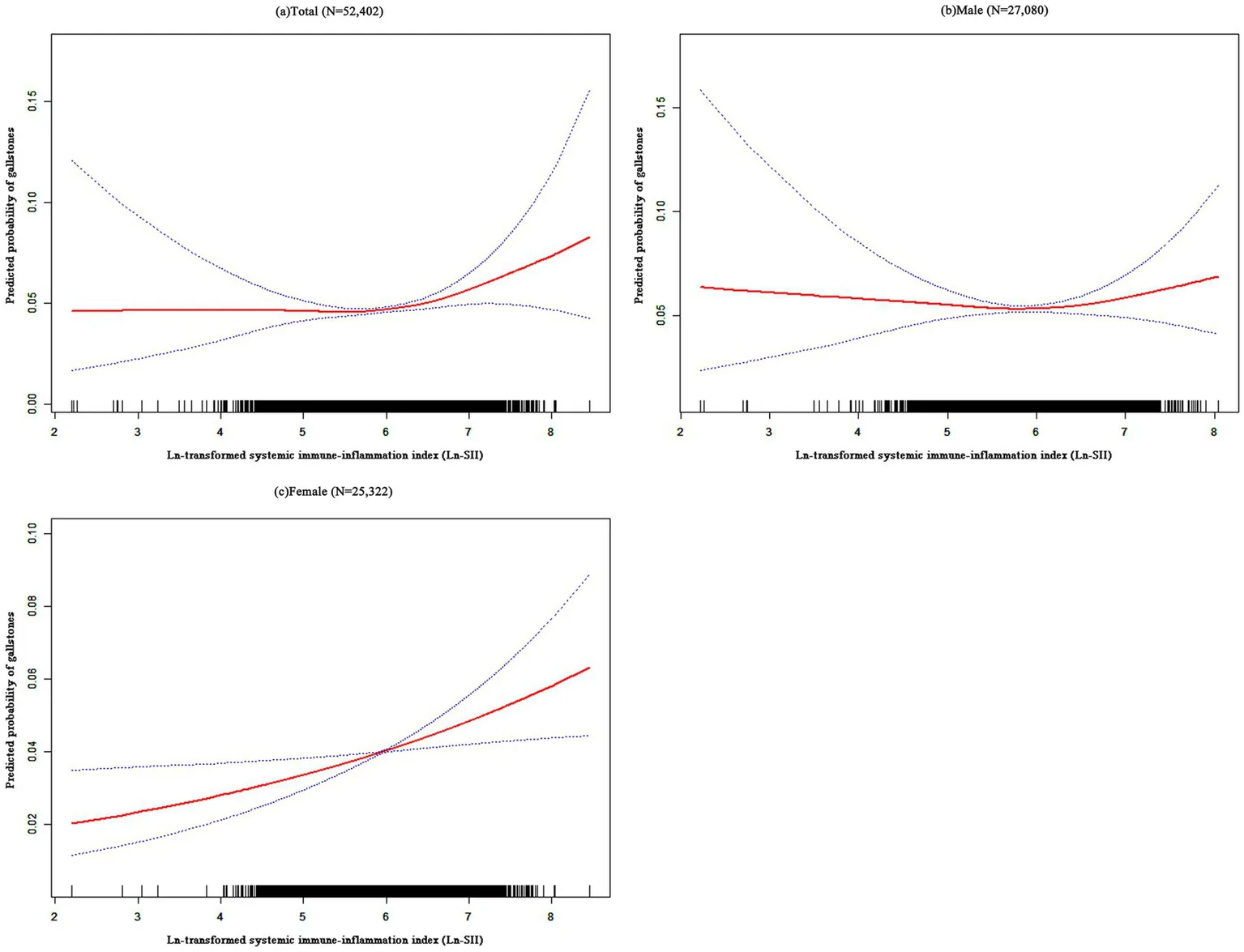
Smoothing spline curves illustrating the association between Ln-SII and gallstone prevalence in the overall population and sex-stratified subgroups. **(a)** Total population (*N* = 52,402); **(b)** males (*N* = 27,080); **(c)** females (*N* = 25,322). Curves were generated using generalized additive models with adjustment for potential confounders. The solid line represents the estimated association, and the shaded area indicates the 95%confidence interval. The curves suggest a potential nonlinear association in the overall population and males, whereas the relationship appeared more linear among females.

Subsequently, we conducted a threshold effect analysis to formally assess the presence of non-linearity using a two-piece wise linear regression model, as presented in Table 5. In the overall population, the inflection point of Ln-SII was identified at 6.50. Below this threshold, the association between Ln-SII and gallstone prevalence was not statistically significant (OR = 1.03, 95% CI: 0.93–1.14, *p* = 0.5996). However, when Ln-SII exceeded 6.50, a significantly stronger association was observed (OR = 1.77, 95% CI: 1.21–2.58, *p* = 0.0033). The log-likelihood ratio test supported the existence of a statistically significant threshold effect (*p* = 0.016). In the male subgroup, a comparable threshold effect was detected, with an inflection point at 6.52. Below this value, no significant association was found (OR = 0.95, 95% CI: 0.82–1.08, *p* = 0.3892),whereas above this threshold, the association became statistically significant (OR = 1.98, 95% CI: 1.12–3.48, *p* = 0.0183), with the log-likelihood test again indicating a significant threshold effect (*p* = 0.025). Among females, the estimated inflection point was 6.39. Below this value, Ln-SII was not significantly associated with gallstone prevalence (OR = 1.15, 95% CI: 0.96–1.36, *p* = 0.1296). Above the threshold, the association showed a positive trend but did not reach statistical significance (OR = 1.53, 95% CI: 0.98–2.38, *p* = 0.0637). Furthermore, the likelihood ratio test did not provide evidence of a significant threshold effect among females (*p* = 0.301), suggesting that the association between Ln-SII and gallstone prevalence did not significantly deviate from linearity in this subgroup.

**Table 5:** Threshold effect analysis of the Ln-SII on the occurrence of gallstone using a two-piece wise linear regression model.

Ln-SII index	OR, (95%CI)	*p-*value
Total population		
Fitting by standard linear model	1.10 (1.01, 1.21)	0.0349
*Fitting by two-piecewise linear model*		
Inflection point	6.50	
<6.50	1.03 (0.93, 1.14)	0.5996
>6.50	1.77 (1.21, 2.58)	0.0033
Log-likelihood ratio	0.016	
Male		
Fitting by standard linear model	1.02 (0.90, 1.15)	0.7913
*Fitting by two-piecewise linear model*		
Inflection point	6.52	
<6.52	0.94 (0.82, 1.08)	0.3892
>6.52	1.98 (1.12, 3.48)	0.0183
Log-likelihood ratio	0.025	
Female		
Fitting by standard linear model	1.21 (1.06, 1.39)	0.0059
*Fitting by two-piecewise linear model*		
Inflection point	6.39	
<6.39	1.15 (0.96, 1.36)	0.1296
>6.39	1.53 (0.98, 2.38)	0.0637
Log-likelihood ratio	0.301	

Sex; BMI; hypertension; diabetes; age; daily exercise; smoking; alcohol drinking; TG; HDL-C; LDL-C were adjusted.

## Discussion

Our study identified a significant association between the systemic immune-inflammation index (SII) and gallstone prevalence in a Chinese adult population. In sex-stratified analyses, different association patterns were observed between males and females. Among males, a nonlinear relationship was identified, with an inflection point at 6.52, beyond which higher SII levels were significantly associated with increased gallstone risk. In contrast, females exhibited a relatively linear positive association between SII levels and gallstone prevalence, without evidence of a significant threshold effect based on the likelihood ratio test. These findings suggest that the relationship between systemic inflammation and gallstone risk may show different patterns across sex strata; however, further studies are needed to validate these observations and clarify the potential biological mechanisms underlying these patterns.

Gallstone disease primarily affects individuals aged over 40 years, with a stronger prevalence observed in females. The development of gallstones is influenced by a combination of genetic, environmental, and metabolic factors. Key risk factors include obesity, insulin resistance, dyslipidemia, and advanced age (25, 26). Our study observed a higher prevalence of gallstones in men, potentially due to regional lifestyle factors, dietary habits, and metabolic conditions such as obesity and insulin resistance, which may predispose males to a greater risk of gallstone formation despite the traditionally higher incidence in females.

The supersaturation of bile with cholesterol represents a core pathophysiological mechanism in gallstone formation, primarily driven by impaired gallbladder motility and altered bile acid metabolism (27). Beyond these metabolic and mechanical disruptions, inflammatory mediators—such as TNF-α and IL-6—play a critical role in modulating bile composition and reducing gallbladder contractility, thereby facilitating pathogenesis (17). These cytokines also contribute to the dysregulation of lipid metabolism, further increasing cholesterol saturation within bile—a key determinant of stone formation (27). Moreover, recent evidence underscores the contribution of systemic inflammation to the progression of gallstone disease. Chronic inflammatory states, commonly associated with metabolic disorders like insulin resistance and obesity, can amplify local inflammatory responses within the gallbladder. This localized inflammation not only impairs contractile function but also compromises the integrity of the gallbladder mucosal barrier, thereby promoting cholesterol crystal nucleation and gallstone development (16). In this context, the SII, an index that reflects systemic inflammation, has garnered attention as a potential biomarker.

In our study, the crude comparison showed no significant difference in Ln-SII between participants with and without gallstones, whereas the adjusted models revealed a positive association. This discrepancy can be attributed to the different statistical information provided by the two approaches. Crude comparison tests a difference in mean Ln-SII between groups, whereas logistic regression estimates the change in the odds of gallstones per unit increase in Ln-SII. In addition, age, sex, and BMI are strongly associated with both SII and gallstone risk, and their opposing or heterogeneous effects may attenuate the crude association. Sex also modifies the SII and gallstone relationship, and combining male and female participants may dilute the overall crude signal. Finally, a single blood measurement and the cross-sectional design may not adequately capture long-term inflammatory status. Therefore, a null crude comparison does not exclude a true association, and adjusted, sex-stratified analyses are necessary to reveal the underlying relationship.

The emergence of a positive association after adjustment further suggests that the inflammatory signal captured by SII may be partly masked by major demographic and metabolic confounders, particularly age, sex, and BMI. After controlling for these factors, elevated SII may reflect a chronic pro-inflammatory state that promotes biliary cholesterol supersaturation, impairs gallbladder contractility, and compromises gallbladder mucosal integrity, thereby facilitating cholesterol crystal nucleation and stone formation (16, 17, 27). The sex-specific patterns observed in our study indicate that hormonal and metabolic differences modulate this relationship. In women, estrogen increases hepatic cholesterol secretion and reduces bile acid synthesis while also regulating immune responses (28, 29); these effects may render women susceptible to gallstone formation even at relatively low levels of systemic inflammation, resulting in a linear association. In men, lower estrogen levels and the potential protective effects of testosterone on lipid metabolism and inflammation (30) may require a higher inflammatory burden before biliary alterations become evident, producing a threshold-like association. Lifestyle and dietary factors that differ by sex in Chinese populations may further modify this relationship. Collectively, these findings suggest that SII may serve as an integrated marker linking systemic inflammation to gallstone risk, with mechanisms that appear to differ by sex and involve both inflammatory and hormonal pathways.

This study has several limitations. First, the cross-sectional design precludes causal inference. Because Ln-SII and gallstone status were assessed at the same time, we cannot determine whether systemic inflammation preceded gallstone formation or whether gallstones themselves induced inflammatory changes. Reverse causation is plausible, as gallstones may cause chronic or subclinical gallbladder inflammation, which could elevate SII. Second, despite adjustment for multiple covariates, residual confounding may remain. We did not have complete data on dietary patterns, physical activity intensity, medication use (e.g., oral contraceptives, hormone replacement therapy, lipid-lowering agents), reproductive history, or socioeconomic status. Third, temporality could not be assessed because SII was derived from a single blood measurement; repeated measurements would be needed to capture long-term inflammatory status. Fourth, gallstones were diagnosed by ultrasound without differentiation of stone type, and asymptomatic cases may have been undetected. Finally, our findings are limited to Chinese adults, and generalizability to other populations requires further validation.

Future longitudinal studies with repeated SII measurements are needed to establish temporality and reduce reverse causation. Studies should also differentiate gallstone types, include detailed dietary and reproductive data, and explore the mechanistic links between specific immune cell subsets and gallbladder pathophysiology. Intervention studies could examine whether reducing systemic inflammation lowers gallstone risk.

## Conclusion

In conclusion, elevated SII is associated with gallstone prevalence in Chinese adults, with sex-stratified patterns. SII may be a useful risk marker, but causal inference is limited by the cross-sectional design.

## Data Availability

The raw data supporting the conclusions of this article will be made available by the authors, without undue reservation.
