# Supplementary material for: Sex-stratified association between systemic immune-inflammation index and gallstone risk: a cross-sectional study in a Chinese adult population
Source: Front Public Health. 2026 Oct 5;14:1923451. doi: 10.3389/fpubh.2026.1923451 (PMC13677865; doi:10.3389/fpubh.2026.1923451)
Supplement: Supplementary file 1 [file Table_1.DOCX]

| Supplementary Table. Generalized additive model (GAM) results for the association between Ln-SII and gallstone prevalence, overall and by sex | | | | | | |
| --- | --- | --- | --- | --- | --- | --- |
| Population | N | edf | Ref.df | Chi.sq | P valye | UBRE score |
| Total | 52,402 | 2.82 | 3.65 | 7.81 | 0.0788 | -0.64 |
| Male | 27,080 | 1.74 | 2.24 | 1.00 | 0.6126 | -0.60 |
| Female | 25,322 | 1.01 | 1.01 | 7.60 | 0.0061 | -0.68 |
